# Prevalence and types of persistent dyslipidemia in patients treated with statins

**DOI:** 10.3325/cmj.2013.54.339

**Published:** 2013-08

**Authors:** Željko Reiner, Eugenia Tedeschi-Reiner

**Affiliations:** 1 Department of Internal Medicine, University Hospital Center Zagreb, University of Zagreb School of Medicine, Zagreb, Croatia; 2 Department of Ophthalmology, University Hospital Center Sestre Milosrdnice, Zagreb, Croatia; 3 Department of Ophthalmology, University of Osijek School of Medicine, Osijek, Croatia

## Abstract

**Aim:**

To determine the prevalence and types of persistent dyslipidemia in patients treated with different statins to reduce cardiovascular disease (CVD) risk, as well as to determine the proportion of high risk patients who did not reach the lipid target values and assess cardiologists' further treatment advice for these patients.

**Methods:**

This cross-sectional, observational study recruited 1849 outpatients from all parts of Croatia between January and September 2011 (44.6% women), 19 to 90 years old (average age 63.13) treated with statins for at least 6 months. We analyzed how the potency and type of lipid-lowering treatment were correlated with CVD risk level and achieving treatment goals according to 2007 Joint European Guidelines on CVD prevention.

**Results:**

Most patients (81.3%) were at high risk for CVD. The most frequently used statin was atorvastatin (42.8%), followed by simvastatin (27.6%) and rosuvastatin (22.8%). Only 35.5% patients achieved low density lipoprotein-cholesterol treatment target. Patients treated with more potent statins had better results. A total of 22.3% of patients had high density lipoprotein-cholesterol below 1.0 mmol/L ( ~ 40 mg/dL) for men and below 1.2 ( ~ 45 mg/dL) for women and 46.4% had triglycerides above 1.7 mmol/L ( ~ 150 mg/dL) but there were no significant differences between statins in improving these parameters. Most of the patients on more potent statins were not advised by their cardiologists to change the type or dosage of statin, which was more common in patients on less potent statins.

**Conclusion:**

A considerable number of patients treated with statins did not achieve the treatment goal values. The results were better in patients treated with more potent statins and cardiologists advised them much less frequently to change the type and dosage of statin. There is a need for more intensive treatment, especially for high-risk patients. This could be accomplished by optimizing patients’ adherence, using more potent statins, titrating current statin therapy to higher doses, or using a combined lipid-lowering treatment.

Dyslipidemia is one of the most important risk factors for cardiovascular disease (CVD). According to epidemiological data in the US, 29.2% of persons aged 45 to 84 years without clinical CVD have dyslipidemia (1). In Croatia, about 80% of persons without CVD have total cholesterol >5.0 mmol/L ( ~ 190 mg/dL), more than 70% have low density lipoprotein (LDL)-cholesterol >3.0 mmol/L ( ~ 115 mg/dL), almost 50% have triglycerides >1.7 mmol/L ( ~ 150 mg/dL), and about 20% have high density lipoprotein (HDL)-cholesterol <1.0 mmol/L ( ~ 40 mg/dL) for men and <1.2 mmol/L ( ~ 45 mg/dL) for women (2). An adequate treatment of dyslipidemia significantly reduces the risk for CVD. Epidemiological studies, as well as angiographic or clinical trials, confirmed that a reduction of total and LDL-cholesterol, must be of prime concern in both primary and secondary prevention of CVD (3,4). It has been shown that 10% reduction of plasma total cholesterol is followed by a 25% reduction of coronary artery disease incidence after 5 years, and that a reduction of LDL-cholesterol for 1 mmol/L is accompanied by a 21% reduction of CVD mortality and non-fatal myocardial infarction (5,6).

The Fourth Joint European Task Force Guidelines on cardiovascular disease prevention in clinical practice (7) defined the target treatment levels for total and LDL-cholesterol and also suggested optimal levels of HDL-cholesterol and triglycerides. Reaching target lipid levels is of utmost importance for reduction of cardiovascular risk.

Statins are widely prescribed for reducing total and LDL-cholesterol and, with much less effect, triglycerides, as well for mildly to moderately increasing HDL-cholesterol (3). There are very few data on the prevalence of statin-treated patients who have achieved lipids treatment goal levels according to Fourth Joint European Task Force Guidelines in Europe and there are no such data for Croatia. Also, there are no data on cardiologists' further treatment decisions for patients who have not achieved target lipids levels.

The aims of this study were to determine the proportion of patients who reached the optimal levels of total and LDL-cholesterol according to the Fourth Joint European Task Force Guidelines on cardiovascular disease prevention in clinical practice after at least 6 months of statin therapy, to determine the proportion of patients with high cardiovascular risk who reached these levels (7), and to determine cardiologists' further treatment advice for these patients.

## Methods

This cross-sectional observational survey included 154 cardiologists from all parts of Croatia who were consecutively selected from the Croatian cardiac society's directory. They were not reimbursed for the participation. The country was divided into five geo-economic regions and a consecutive sample of cardiologists from each region was obtained. If a cardiologist declined, consecutive sampling continued until the required number of cardiologists (at least 50% for each region) was achieved. The sample of cardiologists is a representative one since there were 286 licensed cardiologists in Croatia at the time of the study. The cardiologists consecutively enrolled patients aged 18 years or older who came to consultations and were treated with any statin available in Croatia (rosuvastatin, simvastatin, atorvastatin, lovastatin, and fluvastatin) for at least last six months and without changing the dose for at least the last 4 weeks (up to at least first 10 patients starting from the beginning of the survey). We excluded the patients who had been participating in any clinical interventional study during the previous 12 months. All the data were collected from clinical examinations and medical charts from single outpatient visits between January and September 2011.

Information was collected on the demographic characteristics (age, sex etc), serum lipid profile (total and LDL-cholesterol, HDL-cholesterol, triglycerides), and HbA1c at the time of consultation, the name and daily dosage of the current statin, duration of statin treatment, patients’ and family medical history of diabetes mellitus, and risk factors for CVD.

At the patient’s visit, it was recorded whether patients reached the target levels considering their risk level (high or low risk), as well as their compliance with the dosage during the last four weeks based upon their report at the interview and the cardiologist's advice about further lipid-lowering treatment. According to the Fourth Joint European Task Force Guidelines, patients at high risk were defined as those with pre-existing CVD, diabetes and/or systematic coronary risk evaluation (SCORE)≥5%, and patients at low-risk were defined as those without these conditions or SCORE<5% (5). The study was approved by the Central Croatian National Ethics Committee. All patients signed the informed consent form. Statistical analysis was performed using the SPSS 13.0 package (SPSS Inc., Chicago, IL, USA). We used two-way ANOVA, *t* test, or χ^2^ test as appropriate. *P* value lower than 0.05 was considered significant.

## Results

We analyzed the data of 1811 out of 1849 outpatients. Thirty-eight patients were excluded due to missing data – 12 due to missing data about the date of the blood sampling, 25 due to missing data about the date of change of either dose or statin type, and 1 due to missing data about age. The mean age of patients was 63.13 years (median 64 years) ranging from 19 to 90 years, and 44.6% were women.

Most patients (81.3%) were at high risk for CVD. Of those, 56.9% had pre-existing coronary heart disease (CHD), 14.4% had peripheral artery disease, 13.1% had cerebrovascular disease, and 42.1% had diabetes mellitus.

The most frequently used statin was atorvastatin (42.8% of patients), followed by simvastatin (27.6%), and rosuvastatin (22.8%). Much fewer patients were taking fluvastatin (5.8%) and lovastatin (0.6%), while data for 0.3% of patients were not available.

Only 35.5% patients achieved LDL-cholesterol target level and only 33.4% achieved total cholesterol target level (34.0% and 32.3% of those with high CVD risk respectively). However, 44.4% of patients taking rosuvastatin and 39.3% of those taking atorvastatin reached LDL-cholesterol target value compared to 25.0% of those taking simvastatin or 24.2% of those taking fluvastatin. The results were significant for rosuvastatin vs simvastatin (*P* ≤ 0.0001), rosuvastatin vs fluvastatin (*P* ≤ 0.0001), atorvastatin vs simvastatin (*P* ≤ 0.0001), and atorvastatin vs fluvastatin (*P* ≤ 0.0001). A total of 37.9% of patients on rosuvastatin, 37.1% of those on atorvastatin, 25.7% of those on simvastatin, and 25.7% of those on fluvastatin reached total cholesterol target level. The difference in achieving the total cholesterol target level was significant for rosuvastatin vs simvastatin (*P* ≤ 0.0001) and rosuvastatin vs fluvastatin (*P* = 0.013) but also for atorvastatin vs simvastatin (*P* ≤ 0.0001) and atorvastatin vs fluvastatin (*P* = 0.020).

In patients with high CVD risk, only 34.0% reached LDL-cholesterol target level and only 32.3% reached total cholesterol target level. Of these patients, 42.6% of those treated with rosuvastatin reached the target LDL-cholesterol level, which was more than 36.7% of those treated with atorvastatin, 25.3% of those treated with simvastatin, or 25.0% of those treated with fluvastatin. The difference in achieving LDL-cholesterol target level was significant for rosuvastatin vs simvastatin (*P* ≤ 0.0001) and rosuvastatin vs fluvastatin (*P* = 0.002) but also for atorvastatin vs simvastatin (*P* = 0.002) and atrovastatin vs fluvastatin (*P* = 0.026). The difference in achieving total cholesterol target level was significant for rosuvastatin vs simvastatin (*P* = 0.001) but also for atorvastatin vs simvastatin (*P* ≤ 0.0001).

A total of 22.3% of patients on statins had low HDL-cholesterol and 46.4% had elevated triglycerides (24.0% and 46.8% of those with high CVD risk, respectively). There were no significant differences between different statins in improving these parameters.

**Figure 1:**
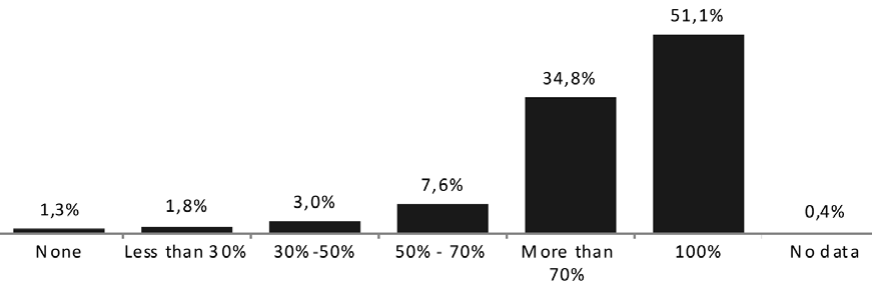
Compliance with the prescribed statin therapy: the percentage of the recommended statin dose taken by the patient in the last 30 days (1811 patients).

There were 51.1% of patients who fully complied with the prescribed dose, 34.8% complied with more than 70% of the prescribed dose, 7.6% with between 50% and 70% of the prescribed dose, 3.0% with between 30% and 50% of the prescribed dose, 1.8% with less than 30% of the prescribed dose, and 1.3% were not taking the prescribed statin at all. For 0.4%, the data were missing (Figure 1).

**Figure 2:**
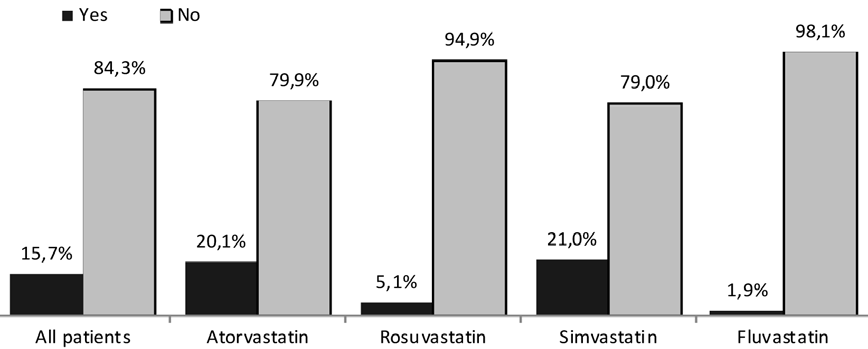
The percentage of patients in whom the statin dose was changed (closed bars) or not changed (gray bars) according to the prescribed statin type (1811 patients).

Concerning the cardiologists' further treatment advice for patients who did not reach target cholesterol levels, 56.2% of patients on fluvastatin and 39.0% of patients on simvastatin were recommended to change the type of statin. This was significantly more than 22.3% of patients on atorvastatin or 18.9% of those on rosuvastatin (*P* ≤ 0.0001). On the other hand, most of patients on rosuvastatin treatment were advised to continue with the same statin without changing the dose (62.2%), while significantly fewer patients on atorvastatin (54.5%), simvastatin (43.6%), and fluvastatin (41.9%) received such advice (*P* ≤ 0.0001). 18.2% of patients taking rosuvastatin and 20.8% of those taking atorvastatin were advised to change the dose of the drug, while 15.8% taking simvastatin and no patient taking fluvastatin received such advice (for rosuvastatin vs simvastatin and atorvastatin vs fluvastatin *P* ≤ 0.0001) (Figure 2).

## Discussion

The present study showed that almost two in three patients on statin treatment in Croatia did not reach the LDL-cholesterol target value recommended by the Fourth Joint European Task Force Guidelines on cardiovascular disease prevention in clinical practice (7). These results are worse than the results of EUROASPIRE III survey performed on 13 935 patients with CHD in 22 European countries. This study used total cholesterol targets of <4.0 mmol/L ( ~ 155 mg/dL) and LDL-cholesterol of <2.0 mmol/L ( ~ 80 mg/dL) and showed that more than two-thirds of high-risk patients had elevated total cholesterol and only one third of patients on lipid-lowering medication achieved the total cholesterol goal, while about half of them achieved LDL-cholesterol goal (8). Croatian patients participating in this study had about the same results. A possible explanation for the differences between the studies might be that the present study analyzed all the patients receiving statins, while the EUROASPIRE III analyzed only the patients with proven CVD.

TASPIC-CRO survey found that 69% of 15 520 Croatian patients with CHD had elevated total and LDL-cholesterol (65% and 68%, respectively in the last part of this survey performed in 2002-2003 – TASPIC-CRO V) despite the fact that 57% of them were taking statins (71% in TASPIC-CRO V) (9). According to the more recently published CRO-SURF survey, although 59% Croatian patients with CHD used statins, 57% still did not achieve target values (10). However, it has to be pointed out that the target value for total cholesterol was changed between TASPIC-CRO V and CRO-SURF from <5.0 mmol/L ( ~ 195 mg/dL) to <4.5 mmol/L ( ~ 175 mg/dL). In CRO-SURF, only 68% of patients achieved the LDL-cholesterol level below 2.0 mmol/L ( ~ 80 mg/dL) and the same percentage achieved the LDL-cholesterol level <3.0 mmol/L ( ~ 115 mg/dL), which was the target value when TASPIC-CRO V was performed. In TASPIC-CRO V, 37% of patients had low HDL-cholesterol and in CRO-SURF study 33%, which is not significantly different. It is difficult to compare these studies with ours, since TASPIC-CRO V was finished almost a decade ago while CRO-SURF included considerably fewer patients and was a pilot study.

Apart from the mentioned studies, which had different aims than our study, there have been only very few cross-sectional and cohort studies aiming to investigate the effectiveness of statin treatment similar to this one and they used somewhat different methodologies and approaches (11-13). The largest one was the recently published DYSIS study, which showed that 48.2% of patients treated with statins from 11 European countries and Canada did not achieve the therapeutic goal for LDL-cholesterol (14,15). Although this was also a disappointing result it is still better than our findings.

On the other hand, the results of our study are similar to the results of A-SACT survey, which found that approximately 70% of CHD patients did not achieve the LDL-cholesterol target of <2.5 mmol/L ( ~ 100 mg/dL) and approximately 94% of those with very high risk did not achieve <1.8 mmol/L ( ~ 70 mg/dL) (16). They are also partially similar to the results of a recently published CEPHEUS pan-Asian survey, which was performed on a very similar population (apart from the racial differences) according to age, male/female ratio, and CVD risk level (17). In this survey, however, the most commonly used statin was simvastatin, followed by atorvastatin and rosuvastatin. In NCEP ATP III (18), the percentage of all patients who achieved their LDL-cholesterol goal was higher than in our study (49.1% vs 35.5%), but patients on rosuvastatin therapy were more likely to achieve target LDL-cholesterol level than those on other statins.

In much older L-TAP survey, the overall LDL-cholesterol rate of patients who reached the target level was 73%, ranging from 47% to 83.5% in different countries (at that time the goal values were much higher), while in a more recent CEPHEUS pan-European survey, which used the NCEP ATP III target values it was 57.4%, which is also much higher than in our study (19,20). If our study had been performed after publishing of the new ESC/EAS guidelines for the management of dyslipidemias and the recently published Joint European Guidelines on CVD prevention in clinical practice with the new LDL-cholesterol targets and new CVD risk stratification, our results would have been even worse (3,21).

When analyzing the frequency of switching to another statin or increasing the statin dose, our results could be compared with those of a German study that analyzed the data from an internet-based registry (22). This study showed that cardiologists recommended switching to another statin in 14.6% patients and increased the dose of statins in 22.9% patients. In our study, fluvastatin was almost exclusively used in patients with only moderately increased LDL-cholesterol and therefore only a few of them needed to change the dose. Due to the best cholesterol-lowering effects of rosuvastatin, 64.2% patients treated with this statin did not need the change of the dose, while 18.2% were advised to change the dose and only 18.9% were advised to switch to other statin.

The results of the present survey indicate that the use of more potent statins, such as rosuvastatin and atorvastatin, results in better LDL-cholesterol lowering. It seems that the reason for not achieving LDL-cholesterol goal in a great number of patients are barriers to translating evidence-based data on lipid target values as recommended in the guidelines into routine clinical practice. The barriers include insufficient physicians’ knowledge of the guidelines, inappropriate statin choice, failure to titrate statin dosage adequately, lack of time and funding, but also lack of patients’ awareness and adherence to lipid-lowering therapy (23-25).

This study indicates that only about half of the patients treated with statins reported that they fully complied with the prescribed dose, while about one third of patients complied with more than 70% of the prescribed dose. Although this does not seem low in comparison with some other surveys, it has to be improved since it has been shown that a high level of compliance with statin therapy (80% or greater) is associated with progressively increasing clinical benefits, both in primary and secondary CVD prevention (26-28). It has been also shown that patients who are very far from reaching serum lipid goals have the highest likelihood of lipid lowering medication non-adherence (29). Barriers to better adherence to statin treatment include the patients’ fear of possible adverse effects, forgetfulness, and the lack of belief in benefits (26). In Croatia, noncompliance could not be explained by drug cost or copayment, which are an important reason for non-adherence in some other countries, since in Croatia generic statins are fully covered by the compulsory health insurance (28).

This study has several limitations. One of them is that since this was a single-point observational study, we used current or retrospective data from patients’ medical records. Lipid parameters were also taken from patients’ medical records and there was no blood sample collection and central evaluation of lipid parameters. Another source of bias may be non-participation, since study participants were included only by the cardiologists who were willing to participate and are therefore likely to be more interested in treating dyslipidemia than average cardiologists.

The results of this study are worrying since they indicate that most patients on statin treatment fail to achieve recommended total and LDL-cholesterol target values and suggest the need for better treatment of dyslipidemia, particularly among high-risk patients. This could be achieved by improving patients’ adherence, choosing more potent statins and applying them in appropriate doses by adequate titration of current statin treatment to higher doses, and the use of combined treatment (30,31,32). These might offer a means to increase the number of patients who meet their lipid target values according to the guidelines.
